# Expression of C-Reactive Protein in Rectal Cancer

**DOI:** 10.4021/gr2009.03.1279

**Published:** 2009-03-20

**Authors:** Paulo de Carvalho Contu, Simone Santana Contu, Mario Antonello Rosito, Luis Fernando Moreira

**Affiliations:** a Post-Graduate Programme in Surgery, Hospital de Clínicas de Porto Alegre University Hospital, Federal University of Rio Grande do Sul, Brazil. Ramiro Barcelos, 2400 - 2° andar, Santa Cecília, Porto Alegre, RS, PO 90.035-930 Brazil; b Division of Coloproctology, Department of Surgery, Hospital de Clínicas de Porto Alegre University Hospital, Federal University of Rio Grande do Sul, Porto Alegre, Brazil. Ramiro Barcelos, 2350 – sl 600, Santa Cecilia, Porto Alegre, RS, PO 90.035-930 Brazil

**Keywords:** C-reactive protein, Rectal cancer, Inflammation, Carcinogenesis, Immunohistochemical expression

## Abstract

**Background:**

The possible involvement of inflammation on colorectal carcinogenesis has potential prognostic, preventive and therapeutic implications. We investigated immunohistochemically whether C-reactive protein is expressed in human primary rectal adenocarcinoma and assessed its relationship with clinicopathological findings.

**Methods:**

Ninety-one rectal cancer samples and 22 normal control samples were immunohistochemically analysed.

**Results:**

Cell accumulation of C-reactive protein was observed in 65 (71%) out of 91 patients with rectal adenocarcinoma and in all 22 control cases (p < 0.01). No significant difference was observed regarding to clinicopathological features or survival rates, but a linear correlation between the positivity proportion of C-reactive protein and Dukes-Turnbull stage (p = 0.005) was observed.

**Conclusions:**

These data suggest that C-reactive protein might play a role in rectal carcinogenesis, but seems not to affect prognosis. Additional studies are warranted in larger population samples.

## Introduction

The involvement of chronic inflammation on colorectal carcinogenesis has been studied for a long time. Inflammatory bowel diseases, such as ulcerative colitis and Crohn’s disease, have been associated with increased risk of colorectal cancer [1], and several studies have suggested a reduced risk of colorectal cancer associated with non steroid anti-inflammatory agents [2-5].

C-reactive protein (CRP) is the prototype of acute phase proteins’ family, which increases in response to infection, trauma, burning, tissue infarction, inflammation and tumours [6]. Its expression has been known to be induced by pro-inflammatory cytokines [7], and it is well known that it is synthesized mainly in hepatocytes. However, there have been some investigations presenting that an excessive elevation of CRP is occasionally found in such patients with liver failure who are requiring liver transplantation [8].

The significance of serum elevation of CRP as an indicator of tumour malignant potential and outcome of patients has been investigated in human gastrointestinal carcinomas [9, 10], renal cell carcinoma [11], ovarian carcinoma [12] and myeloma [13]. However, the role of histological expression of CRP has not been clarified yet and it also remains controversial whether CRP is solely derived from hepatocyte production as a systemic response to inflammatory events or from synthesis and accumulation in tumour cells as a local response to its development. In this study, we investigated immunohistochemically whether CRP is expressed in rectal tumour cells and evaluated its significance and malignant potential in colorectal carcinogenesis.

## Materials and Methods

Tissue samples were collected from 91 patients with primary and sporadic rectal adenocarcinoma surgically resected in the Colorectal Unit, Hospital de Clínicas de Porto Alegre University Hospital. None of the patients received neoadjuvant therapy. There were 51 females and 40 males, age ranged from 19 to 84 (mean of 61) years. Follow-up of the patients was carried out up to 60 months or up to tumour-related death. As a control group, normal histopathological rectal biopsies obtained through colonoscopy from 22 patients were studied. There were 7 males and 15 females with mean age of 49 (range 30 to 75) years. None of the control subjects had any family history of cancer.

Immunohistochemical assessment of the 113 paraffin-embedded tissues was performed using a sheep antihuman polyclonal CRP antibody (Biogenesis Ltd, England, UK) diluted at 1:100. To determine the antibody reactivity, the avidin-biotin peroxidase complex (ABC method; kit LSAB DAKO) was used. Positive cases were identified through visualization of a brownish cellular granulation stained by the diaminobenzidine (DAB) chromogen. As positive controls, samples of liver tissue were used. Reactions conducted without the primary antibody were used as negative controls in liver samples as well. Inflammatory cells depicting immunoreactivity were used as internal positive controls. Cases were identified as positive if more than 10% of tumour cells in a 400 magnification microscopic field showed accumulation of CRP. Two independent pathologists analysed each slide.

The study was performed after approval by the Ethics and Scientific Committees of University Hospital and written informed consent as obtained from all participants.

Variables were described by mean and standard error. Chi-square test and student’s t-test was used to compare clinicopathological data. The cumulative survival rates were calculated by Kaplan-Meier method and significance by logrank. Multivariate analysis was calculated according to Cox’s proportional hazards model in a forward stepwise manner. Statistical significance was considered at p level of 5%. All analyses were calculated using the SPSS 10.0 software.

## Results

**Figure 1:**
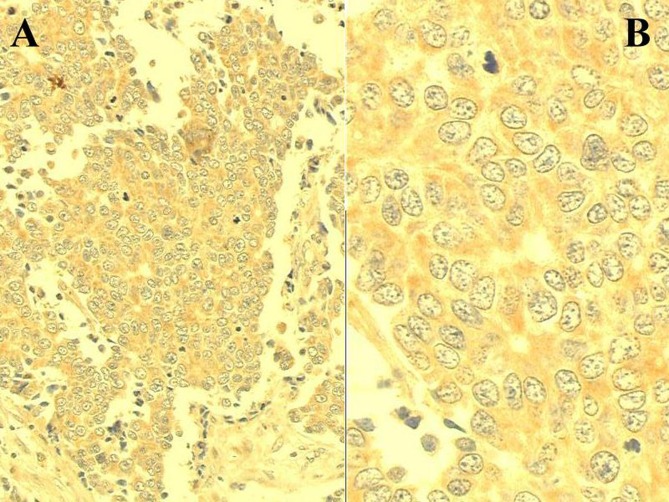
C-reactive protein immunoexpression in a sample of rectal adenocarcinoma. Positive cells display cytoplasmic brownish granulation stained by the DAB chromogen (ABC method; A: x 200; B: x 500).

**Figure 2:**
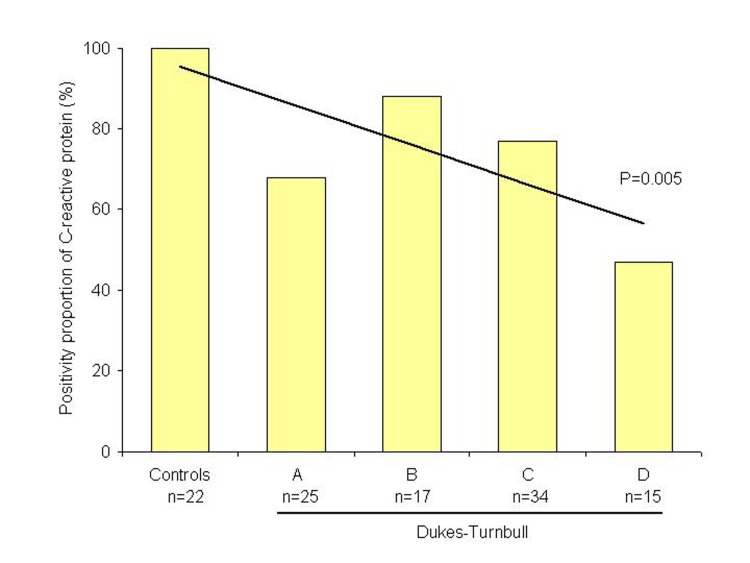
Graphic representation of the positivity proportion of C-reactive protein in rectal tumours (n = 91) compared with controls (n = 22).

Inflammatory cells depicting immunoreactivity were positive cells (Fig. 1). Cellular accumulation of CRP was observed in 65 (71%) out of the 91 patients with rectal adenocarcinoma and in all 22 controls (p < 0.01). Clinicopathological characteristics of patients and controls are shown in Table 1. No significant difference was observed regarding age, sex, tumour location, histological differentiation and tumour depth or lymph node involvement. However, a linear correlation was observed between the positivity proportion of CRP and Dukes-Turnbull stage (p = 0.005; Fig. 2).

**Table 1:** Clinicopathological features of controls versus cases and of deceased cases versus survivors

Features	Controls (n=22)	Cases (n=91)	p value	Deaths (n=42)	Survivors (n=49)	p value
Age (median, SE), years	49.1 (13.4)	60.6 (14.2)	<0.01	62.1 (15.3)	59.4 (13.3)	0.38
Male: Female	7:15	40:51	0.30	22:20	18:21	0.13
Location, from anal verge( mean, SE), cm		6.9 (3.4)	––	6.5 (3.1)	7.1 (3.6)	0.39
Differentiation, n (%)						
well		9 (10)	––	1 (2)	8 (16)	<0.01
moderate		71 (78)		32 (76)	39 (80)	
poor		11 (12)		9 (21)	2 (4)	
Depth of invasion, n (%)						
Submucosa		5 (6)	––	1 (2)	4 (8)	0.07
Muscularis propria		24 (26)		7 (17)	17 (35)	
Adventitia		15 (17)		10 (24)	5 (10)	
Surrounding tissues		47 (52)		24 (57)	23 (47)	
Lymph-node positive cases, n (%)		48 (53)	––	31 (74)	17 (35)	<0.01
		n=89				
Circumferential invasion, n (%)		7 (8)	––	6 (15)	1 (2)	0.04
Dukes–Turnbull						
A		25 (28)	––	5 (12)	20 (41)	<0.01
B		17 (19)		5 (12)	12 (25)	
C		34 (37)		22 (52)	12 (25)	
D		15 (17)		10 (24)	5 (10)	
Adjuvant therapy, n (%)		n=85				
Radiotherapy (RT)		5 (6)	––	4 (10)	1 (2)	0.23
Chemotherapy (CT)		3 (4)		1 (3)	2 (4)	
Combined CT + RT		14 (17)		4 (10)	10 (22)	
No adjuvant therapy		63 (74)		30 (77)	33 (72)	
CRP expression ≥ 10%, n (%)	22 (100)	65 (71)	<0.01	30 (71)	35 (71)	0.99

Data are presented as mean (SE: Standard error), medium (interquartile range: 25th-75th percentile), or counting (percentage).

There were no significant differences between 5-year survival rates in patients with rectal adenocarcinoma expressing CRP as compared to those not expressing CRP. Additionally, a multivariate analysis demonstrated that Dukes-Turnbull tumour staging (p = 0.01) and curative resection (p = 0.02) were found to be independent prognostic factors in patients with rectal adenocarcinoma (Table 2).

**Table 2:** Relationship between clinicopathological features and death in patients with rectal tumours

Features (n)		Death		HR	CI 95%	p value
		n	%			
CRP expression						
≥ 10% (65)		30	46.2	1.31	0.58-2.94	0.51
< 10% (26)		12	46.2	1.00		
Age						
≥ 60 years (55)		27	49.1	1.32	0.66-2.64	0.43
< 60 years (36)		15	41.7	1.00		
Circumferential wedge						
Positive (7)		6	85.7	3.64	1.26-10.51	0.02
Negative (82)		34	41.5	1.00		
Dukes-Turnbull						
A (25)	25	5	20.0	1.00		0.01
B (17)	17	5	29.4	1.21	0.33-4.48	0.78
C (34)	34	22	64.7	4.59	1.72-12.27	0.01
D (15)	15	10	66.7	4.85	1.25-18.77	0.02

Data presented as counts and percentages. HR: *hazard ratio* by a multiple Cox model; CRP: Tumour expression of C-reactive protein as measured by percentage of positive cells; CI 95%: 95% confidence interval. *Cox model including only those subjects (*n*=81, events=37) with thorough observations.

## Discussion

Chronic inflammation has been linked to several solid malignancies, including oesophagus, stomach, liver, pancreas, kidney and prostate cancer. Possible mechanisms by which inflammation may contribute to carcinogenesis include the production of cytokines and growth factors that favour tumour cell growth, induction of cyclooxygenase-2 in macrophages and epithelial cells, and generation of mutagenic reactive oxygen and nitrogen species [14].

The acute phase synthesis of CRP is upregulated by proinflammatory cytokines as interleukin-1, interleukin-6, and tumour necrosis factor, which act as autocrine growth factors for neoplasm [7]. It is known that CRP is synthesized mainly in hepatocytes. Actually, Nozoe et al [15] were the first to demonstrate the immunohistochemical expression of CRP in cells of oesophageal squamous cell carcinoma, suggesting that serum elevation of the protein may be partially derived from immediately production of the substance by the tumour cells as well as by the synthesis into the hepatocytes as a bioresponse to cancer.

Many studies have demonstrated association between CRP and colorectal cancer. Nozoe et al reported increased incidences of liver metastases, peritoneal seedings, microscopic lymph nodes involvement, intravascular invasion and poor prognosis in patients with a preoperatively increased serum CRP level than in those with a negative serum protein level [16]. McMillan et al demonstrated a significantly higher recurrence rate after curative resection of colorectal cancer in patients with an acute-phase response compared to those without acute-phase response [17]. Wigmore et al have showed an increased acute-phase protein response in more than a third of patients presenting colorectal cancer associated with more frequent local tumour invasion, fewer curative resections and higher carcinoembryonic antigen (CEA) concentration, but without showing prognostic significance [9], this was in agreement with Chung’s report [18]. In the study of Nielsen et al, serum CRP was found to be a Dukes independent prognostic variable and was found to identify a subgroup of curatively operated patients at risk for decreased survival [19]. Erlinger et al have reported increased plasma concentrations of CRP among individuals who subsequently develop colon cancer, supporting the hypothesis that inflammation is a risk factor for the development of colon cancer in average-risk individuals [20].

However, all of these investigations studied serum CRP, which represents systemic inflammatory response. Moreover, plasma CRP may suffer influence of other variables, such as inflammatory and infectious diseases, collagenosis, cardiovascular events and some medications. To the best of our knowledge, till now, there is no reported investigation on the immunohistochemical expression of CRP in human colorectal tumours. The rationale to perform this investigation is that an immunohistochemical examination would demonstrate whether rectal tumours do express CRP and if so, it would represent the local inflammatory reaction to tumoral invasion.

Cell expression of CRP was observed in 65 of the 91 patients with rectal adenocarcinoma and in all control samples. These data confirm the hypothesis that CRP can be found in other tissues, including neoplasm, beyond the liver. The distribution was significantly different between cases and controls (71% vs 100%), indicating the influence of inflammation in colorectal carcinogenesis. However, these results are opposite to those demonstrated in researches that studied serum CRP [9, 16-20]. An explanation would be that cellular CRP indicates the immediate inflammatory response to tumour invasion, while its serum expression indicates the later systemic reaction.

It was not demonstrated, in the current study, association of CRP expression and prognosis, but a linear trend test indicates correlation of decreasing expression of CRP between controls and Dukes’ stages, and therefore, these findings may suggest a CRP role in tumour progression, mainly at early stages. Indeed, in immunohistochemical analysis of CRP among controls, an intense expression of CRP was observed in the mucus of the cells. This feature was absent or lacking intensity in neoplastic tissues, which may indicate a modified capacity or function in the production and release of CRP. Hypothetically, this may suggest that CRP may play a hole of a mucosal protective factor which deteriorates with tumour progression. However, the lack of prognostic significance due to a small or inadequate sample to determine differences among subgroups should be also considered.

These data suggest that local inflammation, represented by CRP, may play a role in rectal carcinogenesis, but it seems not to affect prognosis. It would be important to carry out this study further with a larger sample, including adenomas, and correlating other inflammatory markers expression with plasma levels in order to examine the hypothesis of cellular contribution to serum levels, effect of the inflammation in colorectal carcinogenesis and to reassess this association with prognosis.
